# Chronic Pediatric Pain and Mental Illness During the COVID-19 Era: A Case Series From Inpatient Child Psychiatry Unit

**DOI:** 10.7759/cureus.20032

**Published:** 2021-11-30

**Authors:** Ankit Jain, Sage Gee, Srikrishna V Malayala, Christopher W Laboe

**Affiliations:** 1 Psychiatry & Behavioral Health, Penn State College of Medicine, Hershey, USA; 2 Internal Medicine, Temple University Hospital, Philadelphia, USA

**Keywords:** covid-19, psychodrama and group therapy, cognitive behavioral therapy, intervention and psychotherapy for children and adolescents, childhood anxiety disorder, depression, chronic pain management

## Abstract

Chronic pain is defined as pain that persists following tissue injury or disease processes and is believed to have taken place after healing. It is a functional impairment that causes discomfort and leads to the inability to perform various essential daily activities. Chronic pain in pediatrics can be of various types, the most commonly prevalent types being headaches, abdominal pain, and limb pain. Adolescents with chronic pain have been found to have generalized anxiety scores twice that of the average population, along with significant impairment of family dynamics. Some forms of chronic pain respond effectively to medication and psychotherapy, and recurrence is common when stress is triggered by external or environmental factors, most recently in the form of COVID-19. In this case series, we discuss three cases of worsening mental health presentations and chronic pain exacerbation in the context of the COVID-19 pandemic. We talk about the unique perspective of chronic pain in the pediatric population and comorbid mental illnesses and their management from a psychiatric standpoint.

## Introduction

Chronic pain is defined as pain that persists following tissue injury or disease processes and is believed to have taken place after healing [1]. It is a functional impairment that causes discomfort and leads to the inability to perform various essential daily activities [2]. Chronic pain treatment aims to achieve significant decreases in pain intensity to help attain an individual's goal to achieve a manageable level of pain to be able to have a good quality of life [2]. Approximately 13.5%-47% of the US population struggles with chronic pain, leading to a cascade of economic and medical problems within our society [3]. Chronic pain in pediatrics can be of various types, the most commonly prevalent types being headache (23%), abdominal pain (22%), and limb pain (22%) [2]. Adolescents with chronic pain have been found to have generalized anxiety scores twice that of the average population, along with significant impairment of family dynamics [2,4]. In recent times, there has been an upsurge in psychiatric presentations such as post-traumatic stress disorder (PTSD), major depressive disorder (MDD), anxiety disorders, and obsessive-compulsive disorder (OCD) since the onset of the COVID-19 pandemic [5-7]. There has been an increase in neuropsychiatric complications following COVID-19 infection across all age groups [8]. Chronic pain has often been noted to be one of the comorbidities in patients with psychiatric illness. Some forms of chronic pain respond effectively to medication and psychotherapy, and recurrence is common when stress is triggered by external or environmental factors, most recently in the form of COVID-19 [9].

In this case series, we discuss three cases of worsening mental health presentations and chronic pain exacerbation in the context of the COVID-19 pandemic. We talk about the unique perspective of chronic pain in the pediatric population and comorbid mental illnesses and their management from a psychiatric standpoint.

## Case presentation

Case 1

A 16-year-old White female with a history of depression and post-traumatic stress disorder presented to the inpatient psychiatry unit with worsening suicidal ideations from social isolation since the COVID-19 pandemic began, along with stressors of argument with their parents. Her home medications were topiramate 50 mg at bedtime for chronic headaches, fluoxetine 40 mg/day for depression, and prazosin 1 mg at bedtime for nightmares secondary to PTSD. No other significant medical or surgical history was noted, and the baseline laboratory results were within normal limits. She reported that she experienced chronic nausea and tension headaches that led to difficulty sleeping. She spent several hours per day lying in bed because that was the only way she found relief. She would often wake up at night and have difficulty falling back asleep in the context of having headaches. The patient was given the Epley and Dix-Hallpike maneuver to rule out the neurological causes of the tension headache and nausea. Treatment options were discussed. Topiramate dose was increased to 100 mg at bedtime and had primarily been successful in addressing her tension headaches and nausea over the course of several weeks with some improvement. The patient was also started on amitriptyline 25 mg at bedtime and was gradually titrated to 50 mg at bedtime, leading to further improvement in her chronic headaches. The patient was also educated on chronic pain and was taught coping skills such as deep breathing, guided imagery, and going for walks to distract herself from focusing on her chronic pain. The patient noted significant improvement in her headaches, depression, and anxiety with medication adjustments and using the coping skills that she learned on the unit. The patient was discharged to a residential treatment center in the context of complex psychosocial needs with appointments set up for psychiatry and therapy.

Case 2

The patient is a 17-year-old transgender female-to-male patient with a history of PTSD, depression, and borderline personality traits. The patient had a history of one previous hospitalization for concerns related to self-harm urges and suicidal ideations. The patient has a history of type 1 diabetes with well-controlled blood sugars on their insulin regimen; no other medical history was noted. The patient reported worsening of depression and feeling that her mood had declined over the past year since the pandemic hit. The patient reported issues surrounding chronic abdominal pain and had not reported this to her pediatrician or a pain physician. The patient was initially started on duloxetine 60 mg/day to address depression and issues with chronic abdominal pain, which was gradually titrated up to 90 mg/day. It was considered to have a trial of SSRI and TCA medication, but the patient reported having a poor response to previous SSRI trials (sertraline and fluoxetine at therapeutic dosages), and TCAs were not considered a safe option given the prior history of an overdose in the patient. Significant improvement in the patient's report of chronic abdominal pain was noted when the dose of duloxetine was titrated up to 60 mg/day. The patient reported improvement in her appetite and improved participation in the milieu, which was noted within two weeks of titrating the dose of duloxetine. The patient continues to await placement in our inpatient unit for placement at a community residential rehabilitation home.

Case 3

The patient is a 13-year-old White female with a history of Ehlers-Danlos syndrome (EDS) type 2 and generalized anxiety disorder who presented with worsening anxiety and depression in the context of deteriorating school grades and getting bullied at school. It was noted that the patient had previously seen a pediatric rheumatologist for her chronic pain issues and was diagnosed with juvenile fibromyalgia. The patient reported having had a previous suboptimal trial of duloxetine with suboptimal pain and depression relief. On having a detailed discussion, consent was obtained to initiate a trial of sertraline at a dose of 25 mg/day, which was gradually titrated to 100 mg/day. The patient and parent were educated on evidence of the role of psychotropic medication in addressing pediatric chronic pain apart from them being helpful in anxiety and depression. No family history of mental health issues was reported, and baseline laboratory results were noted to be within normal limits. The patient and parent were educated on the need for medication compliance, and a referral was made for a therapist who specializes in cognitive behavioral therapy for complex medical needs. The patient was already aware of specific coping strategies such as guided imagery and acceptance and commitment therapy for chronic pain issues. The patient was stepped down to a partial hospitalization program with scheduled follow-up for outpatient psychiatry.

## Discussion

The cases presented here highlight the scenarios of chronic pain worsening along with psychiatric comorbidities in the setting of identifiable stressors, with the COVID-19 pandemic being the common denominator in all of these cases. While there have been more studies on chronic pain treatment options in adults, there has generally been a scarcity of such studies in the pediatric population. In the above cases, significant improvement in mental health symptoms and chronic pain of patients were noted when pharmacological and psychotherapeutic interventions were utilized. In terms of pharmacological management, the evidence of using non-opioid pharmacological agents has been limited mostly to the use of NSAIDs and anticonvulsants, with only recent evidence supporting the use of psychotropic medications, mainly from the antidepressant category [10]. Antidepressants work through pathways that involve increased norepinephrine and serotonin in the synaptic clefts near the spinal cord that have an inhibitory effect on the perception of pain [11]. Anticonvulsants, which are often used as an off-label medication for the treatment of chronic pediatric pain, are believed to work by decreasing the firing frequency of neurons. This can occur through increasing GABA or stabilizing the neurons through channels such as ion channels involving calcium [12]. When utilizing different psychopharmacological options, clinicians need to be mindful of drug-drug interactions and the varied pharmacodynamic impacts of these drugs [13,14].

Chronic pain management in the pediatric population needs a multidisciplinary approach, which requires a combination of medication management, psychotherapy, physical therapy, and relaxation exercises. Many non-pharmacological measures are also considered in managing pediatric chronic pain, including psychosocial factors, physical activity, academia, and parental care [15]. Multimodal approaches to pediatric pain management have been shown to have reduced side effects compared with a single method. In addition, interdisciplinary approaches have been shown to be the most effective for managing chronic pain [16]. We also found that learning coping skills such as guided imagery, progressive muscle relaxation, and mindfulness-based learning were extremely helpful in managing chronic pain in our patients.

Since the COVID-19 pandemic, there has been a rapid shift from in-person visits to telemedicine appointments to a decrease in in-person visits without interruptions in the continuity of care [9,17]. While it is not the optimum solution, it helped bridge the gap because of the scarcity of in-person appointments in pain clinics. A systematic review from 2019 analyzed virtual delivery of cognitive behavioral therapy (CBT) for children and adolescents with chronic pain. According to the study, there was insufficient evidence that CBT provided remotely could significantly reduce chronic pain symptoms. Adolescents exhibited a short-term reduction in pain, but the proof of a long-term decline in pain symptoms was equivocal [18]. There were short-term pain decreases with headaches, but the results did not last for more than at least three months. However, wait times for pain clinics were reported to be around 197.5 days, and virtual CBT might allow teenagers to receive psychiatric care geared toward the management of their chronic pain more quickly and at a reduced cost [19]. In an Internet-based setting, acceptance and commitment therapy has shown favored efficacy in treating child and adolescent chronic pain and following related domains, including depressive symptoms and psychological inflexibility [20]. Virtually delivered care is more widely adopted by the pediatric pain management community, and tools are becoming increasingly available in recent years. Still, the specific treatments may need tailoring to address particular challenges in the context of the current pandemic. In the midst of a shortage of in-person pediatric chronic pain appointments with exceptionally long waiting periods, because of the scarcity of pediatric pain specialists, it is important for psychiatrists and primary care providers to help bridge this gap. With the recent evidence supporting the use of commonly prescribed psychiatric medications for the treatment of chronic pain along with pediatric anxiety and depression, it helps the clinicians make more evidence-based treatment recommendations to their patients and their families.

## Conclusions

This case series highlights the importance of effectively treating chronic pain and mental disorders by the combined therapeutic approach of pharmacology and psychotherapeutic techniques especially in the presence of a significant stressor such as the COVID-19 pandemic. The shift toward virtual healthcare services in the time of social distancing raises concerns about how effective these modalities are compared with traditional services. These concerns have been addressed in randomized controlled trials, which have shown virtual services to deliver various treatments, including psychotherapy, effectively. More robust future studies need to be conducted to see if these effects are long-lasting and can replace or substitute the needs for in-person patient visits for treatment options such as Internet-based cognitive behavioral therapy around the management of chronic pediatric pain. All clinicians should be mindful of obtaining a detailed medical history that includes a history of chronic pain. Interdisciplinary management is the key to providing effective and safe management of chronic pediatric pain. Judicious use of psychotropic medications is an important tool that is readily available to most psychiatrists to manage and treat psychiatric illnesses with comorbid chronic pain conditions.
